# Job satisfaction at older ages: A comparative analysis of Hungarian and German data

**DOI:** 10.1007/s00391-019-01547-x

**Published:** 2019-04-26

**Authors:** Éva Berde, Mariann Rigó

**Affiliations:** 1 Corvinus University of Budapest, Fővám tér 8, 1093 Budapest, Hungary; 2 Institute of Medical Sociology, Medical Faculty, University of Düsseldorf, Universitätsstraße 1, 40225 Düsseldorf, Germany

**Keywords:** Early retirement, Pension reforms, Demographic change, Working conditions, European Working Conditions Survey, Frühverrentung, Renteneformen, Demografischer Wandel, Arbeitsbedingungen, Europäischen Erhebung über die Arbeitsbedingungen

## Abstract

**Background:**

As a response to population aging, reforms to increase the statutory retirement age and closing options for early retirement have been introduced in many European countries. This study analyzed the job satisfaction of employees in two countries with markedly different speeds of pension reforms. The German reform started in 1992 and abolished almost all options of early retirement. The Hungarian reforms started later and were completed only by 2011. Therefore, it is expected that older Hungarian workers were initially more satisfied with their jobs than similarly aged German workers.

**Objective:**

The hypothesis was tested that older workers in a regulatory environment with accessible pathways to early retirement are on average relatively more satisfied with their job than older workers in a country with few and financially less advantageous options for early retirement.

**Material and methods:**

This study used data from the European Working Conditions Surveys. Waves 2005 and 2010 represent years when early retirement pathways were abolished in Germany, while the Hungarian system offered a variety of pathways for early retirement. This is not the case in 2015 having tight regulations in both countries. Logit regressions were estimated using job satisfaction as an dependent variable and a variety of control variables were introduced step by step.

**Results:**

The results from 2005 and 2010 indicate that older Hungarian employees are relatively more content with their job than similarly aged German workers. In 2015 this trend was reversed.

**Conclusion:**

It would be crucial to provide the opportunity and appropriate working conditions for older employees to work if they voluntarily opt for working longer. They seem to be an especially motivated pool of employees, and could productively contribute to decreasing the financial burdens caused by the demographic changes.

**Electronic supplementary material:**

The online version of this article (10.1007/s00391-019-01547-x) contains supplementary material, which is available to authorized users.

## Zusammenfassung

**Hintergrund:**

In vielen europäischen Ländern wurden Reformen zur Erhöhung des gesetzlichen Rentenalters eingeführt und Möglichkeiten der Frühverrentung abgeschafft. In diesem Beitrag werden zwei Länder analysiert, in denen die Renten- und Arbeitsmarktpolitik mit deutlich unterschiedlicher Geschwindigkeit reformiert wurde. Durch die 1992 beginnende deutsche Reform wurden fast alle Möglichkeiten der Frühverrentung abgeschafft. In Ungarn fanden die Reformen später statt und wurden erst 2011 abgeschlossen. Daher steht zu erwarten, dass ältere ungarische Arbeitnehmer eine höhere Arbeitszufriedenheit aufweisen als deutsche.

**Fragestellung:**

Die Hypothese, dass ältere Arbeitnehmer in einem regulatorischen Umfeld mit Möglichkeiten der Frühverrentung relativ zufriedener mit ihrer Arbeit sind als ältere Arbeitnehmer in einem Land mit wenigen, finanziell kaum vorteilhaften Möglichkeiten zur Frühverrentung wurde untersucht.

**Material und Methoden:**

Es wurden Daten aus der Europäischen Erhebung über die Arbeitsbedingungen verwendet. In den Jahren 2005 und 2010 waren die Frühverrentungsregelungen in Deutschland bereits abgeschafft, während das ungarische System weiterhin eine Vielzahl an Möglichkeiten bot. Dies hat sich im Jahr 2015 in beiden Ländern durch strengere Regelungen geändert. Die Logit-Regressionen unter Verwendung der Arbeitszufriedenheit wurde als abhängige Variable geschätzt.

**Ergebnisse:**

Die Ergebnisse von 2005 und 2010 zeigen, dass ältere ungarische Arbeitnehmer relativ zufriedener im Job sind als deutsche Arbeitnehmer der selben Altersgruppe. Im Jahr 2015 ist der Trend entgegengesetzt.

**Schlussfolgerung:**

Es ist wichtig, älteren Arbeitnehmern Möglichkeiten und angemessene Arbeitsbedingungen zu bieten, um zu arbeiten, wenn sie sich freiwillig für eine Verlängerung der Erwerbstätigkeit entscheiden, da sie oft über eine hohe Motivation verfügen und produktiv zur finanziellen Entlastung beitragen können.

## Introduction

Most developed countries face problems due to population aging, and the aging process is expected to be more and more prevalent in developing countries as well [3]. The financial and social problems due to population aging were partly exacerbated by the generous pay as you go pension regulations, in which pensions of the retirees were financed by the earnings of the working population. As the demographic shift advances, the proportion of wage earners relative to beneficiaries is shrinking [10] and pension payments can no longer be covered given the previously set retirement ages and early retirement opportunities.

Recently, policy changes have been introduced in many European countries including reforms to increase the statutory retirement age and closing options for early retirement [15, 21]. This article examines the relationship between pension regulations and older workers’ job satisfaction. Specifically, the job satisfaction of German and Hungarian older workers is compared. Job satisfaction is one of the key determinants of the willingness to work at older ages [22] and besides regulatorily set retirement ages, the most important factor influencing older workers’ labor market activity.

Germany and Hungary were selected for analysis as both countries implemented widely available early retirement schemes starting from the 1970s resulting in low employment rates among the older people; however, they diverged regarding the timing of their activation policies. While early retirement pathways were mostly closed by 2000 in Germany, the Hungarian reform was completed only by 2011. Therefore, comparing Germany and Hungary provides a good example of countries with similar institutional elements in their pension policies before 1990, similar reform steps but the timing of these reforms was markedly different.

The 2005 and 2010 waves of the European Working Conditions Surveys (EWCS) cover a period when retirement rules were more stringent in Germany than in Hungary leaving less options for German older workers to leave the labor market before the official retirement age compared to similarly aged Hungarian employees.^1^ This is not likely to be the case in 2015. After 2011, pension regulations also became stricter in Hungary, captured by the 2015 wave of the EWCS. The article tests the hypothesis that older workers in a regulatory environment with accessible pathways to early retirement are on average relatively more satisfied with their job than older workers in a country with few and financially less advantageous options for early retirement.

It is expected that in 2005 and 2010 older employees in Hungary who work more or less voluntarily have a more positive attitude towards their job relative to prime-aged workers and therefore, higher job satisfaction than similarly aged German employees. This is not likely to be the case in 2015. The findings are in line with the basic hypotheses. The results emphasize the importance for allowing older persons to decide on voluntarily staying in the labor market even if they should or could retire. The results also emphasize the importance of certain company-level practices, which provide appropriate circumstances for older employees to become more satisfied with their job.

Section 2 presents a short overview of the German and the Hungarian pension systems. Section 3 describes the data and the model specifications. The results are discussed in Section 4. Section 5 summarizes the most important policy conclusions.

## German and the Hungarian pension system

In the 1970s both Germany and Hungary relied on a generous pay-as-you-go pension scheme, although based on different ideological and political backgrounds. The German system relied on the egalitarian principles of the Bismarckian pay-as-you-go social security system [4], while the Hungarian one was based on the principles of the paternalist socialist state, with pensions made available on a universal basis from 1975 onwards [1]. In both countries early retirement became a standard instead of being an exception and was applied as a common tool by firms to adapt to economic stagnation [5, 8]. This practice was especially pronounced in Hungary after the regime change and often used as a reaction to the depreciation of a variety of labor market skills [17, 20].

Germany started its reform in 1992 by increasing the statutory retirement age and closing almost all options for early retirement; however, the reforms in the 1990s were contradictory as although several early retirement pathways were closed, new ones were opened. From 2000 onwards the German reforms included further restrictions, such as gradually raising the statutory retirement age and basically all pathways of early retirement were abolished [14, 15]. The change in Hungary started later, in 1996, and was initially less consequent. A variety of early retirement pathways, e. g. disability pensions, unemployment benefit and early retirement provisions, were offered and were mostly used to cushion the decreasing employment opportunities. Disability pensions represented a popular form of early retirement, and were automatically switched into old age pension after reaching the statutory retirement age [21]. The tightening of the regulations started at the beginning of the 2000s but early retirement pathways were still available and the statutory retirement age was on average 2 years lower than in Germany. The reform was completed by closing almost all early retirement pathways only by 2011 [9, 11].

## Data and model specification

Recently, there has been growing interest in analyzing the relationship between job satisfaction and age. Previous literature suggested that job satisfaction is influenced by various factors, e. g. individual characteristics, working conditions, work environment, individual health status and family background. The regressions include a rich set of demographic and firm-level control variables, similarly to previous studies [6, 16, 24]; however, the novelty of this study lies in highlighting the role of pension regulations in retirement decisions through analyzing job satisfaction, an important factor in the decision to exit the labor market. A key policy implication of the calculations, also pointed out previously by Lain [18] and Lain and Vickerstaff [19] emphasizes the need to let and possibly reward motivated workers to stay longer on the labor market.

This study compared the job satisfaction of older employees relative to the prime aged, between Germany and Hungary. Focusing on relative job satisfaction has the advantage of filtering out general age-independent country-specific differences.

To carry out the analysis three consecutive waves of the EWCS were used. This provides internationally comparable cross-sectional data for a large number of countries and, to our knowledge, has not been used previously to analyze the relationship of job satisfaction and age. It includes rich information on employees’ job and demographic characteristics in addition to offering information on job satisfaction. The EWCS has been carried out every 5 years since 1990. The target sample size in Hungary and in most countries was 1000; Germany is one of the exceptions with target sample sizes of 2000. Although EWCS is cross-sectional and thus, does not follow employees over time, it is a suitable tool to compare on average the relative job satisfaction (older vs. prime aged) of Hungarian and German employees. The comparison of average relative job satisfaction and the interpretation of the between-country relative job satisfaction gap rely on two important assumptions. First, it is assumed that there are no country and age-specific systematic differences between the job satisfaction of Hungarian and German workers (note that age-independent, country-specific differences are controlled for). Second, it is assumed that differences in pension regulations constitute the most important difference between the countries, which might have a differential impact on older workers compared to younger. The analysis is based on the 2005, 2010 and 2015 waves of EWCS. The years of 2005 and 2010 are characterized by similar pension regulatory environments within each country. Therefore, to improve the precision of estimates the data of those waves were pooled. On the other hand, wave 2015 should be analyzed separately as the Hungarian regulatory environment experienced profound changes after 2011. The dependent variables include responses to the following questions: “Are you satisfied with your main job?” and “Does your job give you the feeling of work well done?”. While the first question captures the overall job satisfaction of employees and as such may include subjective opinion on many dimensions of the work as well as individual characteristics (e. g. general work attitude, health status) and general well-being, the second question focuses primarily on satisfaction from carrying out the job itself and is less likely to be affected by other external and individual, psychological attributes.

Ordered logit regressions were estimated using two measures of job satisfaction as dependent variables and introducing a variety of control variables step by step. The final specification includes 27 different control variables on demographic features, working environment and job characteristics. Employees are grouped into three age categories: dummy variables for being aged below 30 years (young), aged between 30 and 55 years (prime-aged) and aged over 55 years (older) are used in our regressions.^2^ Interactions between the country and the age dummies are introduced to allow for the possibility that the job satisfaction-age profiles vary between countries; however, the rest of the coefficient estimates are restricted to be the same in both countries. The regressions use the pooled sample of German and Hungarian workers including first the waves 2005 and 2010 (period 1), followed by the analysis on wave 2015 (period 2).

Previously, job satisfaction-age profiles have been estimated both with health measures [7, 12] and without [2, 23]. As information on health status is only available in the 2010 and 2015 waves of the EWCS, including it in all specifications would lead to a large loss of observations and might result in imprecise estimates; however, to get an idea about the influence of health on job satisfaction, the final detailed specification is presented using data on wave 2015 with and without health measure. The regression results including a description of the model specification can be found in the electronic supplementary material.

## Results

**Fig. 1:**
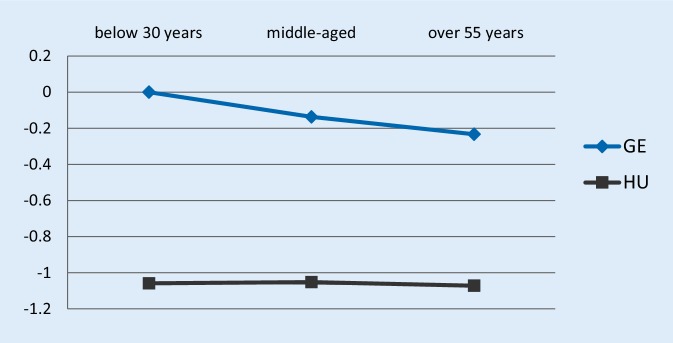
Job satisfaction-age profile estimates of the ordered logit specification using the waves 2005 and 2010, dependent variable: are you satisfied with your main job? *GE* Germany, *HU* Hungary; the Y-axis shows the estimated coefficient of employees’ job satisfaction relative to German young workers

**Fig. 2:**
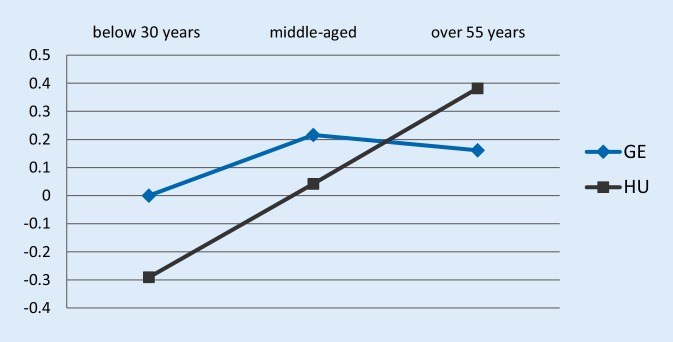
Job satisfaction-age profile estimates of the ordered logit specification using the waves 2005 and 2010, dependent variable: does your job give you the feeling of work well-done? *GE* Germany, *HU* Hungary; the Y-axis shows the estimated coefficient of employees’ job satisfaction relative to German young workers

Figs. 1 and 2 present the estimated job satisfaction-age profiles in the case of both dependent variables, both countries. German young workers serve as the reference category. Therefore, all parameter estimates should be interpreted as relative to German young workers. As shown by Fig. 1, in the first period (waves 2005 and 2010) German employees had a higher level of overall job satisfaction over the entire age profile than comparable Hungarian workers. This is in line with previous empirical results [23]; however, when comparing the job satisfaction of older employees relative to their prime-aged colleagues, Hungarians fared better. There was no significant difference between the job satisfaction of older Hungarians relative to prime-aged, while German older employees were significantly less satisfied than their younger colleagues. The difference between the Hungarian and German older vs. prime-aged relative job satisfaction estimates was also significant at 10% level.

Turning to the results with the “work well-done” dependent variable (Fig. 2), the higher relative job satisfaction of Hungarians was more pronounced. The job satisfaction-age profile in the case of Hungary was upward sloping, and Hungarian older workers outperform German older employees even in absolute terms. The relative job satisfaction of German older workers was not significantly different from the prime-aged employees, while Hungarian older workers were significantly more content with their work compared to their middle-aged colleagues. The t‑test for significance comparing the across country relative job satisfaction estimates indicated significant differences between the Hungarian and German estimates. In sum, the results including waves 2005 and 2010 of the EWCS were in line with the hypothesis suggesting that Hungarian older workers were a positively selected sample, and were more likely to represent a motivated pool of employees willing to work at older ages. On the other hand, German older workers faced with more stringent pension regulations had less option to exit the labor market, therefore, they constituted a more representative sample of employees regarding their motivation and willingness to work. Turning to the results on the 2015 wave of the EWCS, a different picture emerged. Supporting the hypothesis, the Hungarian job satisfaction-age profiles had an inverse U‑shape in the cases of both dependent variables, while the German estimates were similar to those in period 1.

**Fig. 3:**
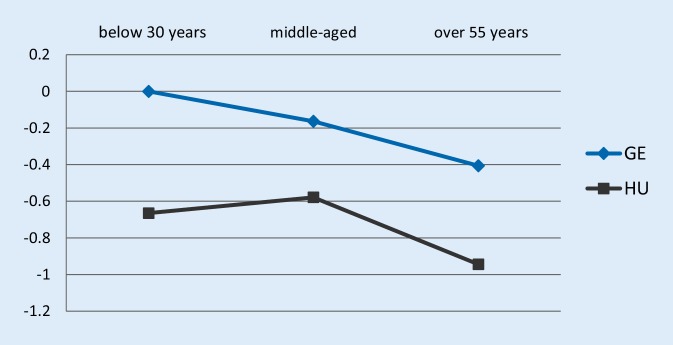
Job satisfaction-age profile estimates of the ordered logit specification using the 2015 wave, dependent variable: are you satisfied with your main job? *GE* Germany, *HU* Hungary; the Y-axis shows the estimated coefficient of employees’ job satisfaction relative to German young workers

**Fig. 4:**
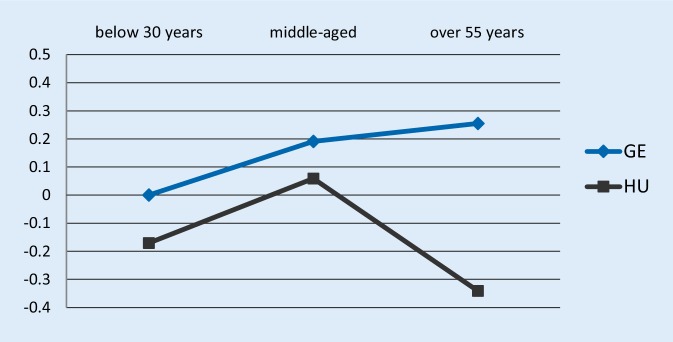
Job satisfaction-age profile estimates of the ordered logit specification using the 2015 wave, dependent variable: does your job give you the feeling of work well-done? *GE* Germany, *HU* Hungary; the Y-axis shows the estimated coefficient of employees’ job satisfaction relative to German young workers

As illustrated by Figs. 3 and 4, Hungarian older workers seemed to be the least satisfied, both compared to their Hungarian younger colleagues or German workers. The difference between the Hungarian and German relative job satisfaction estimates was significant. The gap between the estimates turned into the opposite direction compared to period 1 estimates: in 2015 German older employees were more satisfied than their Hungarian counterparts both in absolute and relative terms.

These results suggest that in a regulatory environment where Hungarian older employees chose more or less voluntarily to work at older ages, the surveyed pool of Hungarians showed a higher level of job satisfaction. On the other hand, in a regulatory environment with strict retirement rules, older employees had almost no room for maneuver to retire early, which is reflected by the job satisfaction responses. Similarly to the analysis on period 1 data, the results rely on the assumption that the only country-specific changes between period 1 and period 2 having a differential impact on older and younger workers are due to changes in the pension regulations. It is believed that most of the changes from 2010 to 2015 are gradual and no comparable sudden change affecting the job satisfaction of older and younger employees differently took place. To our knowledge, in the examined period no other radical policy changes have been introduced in the Hungarian economy.

**Fig. 5:**
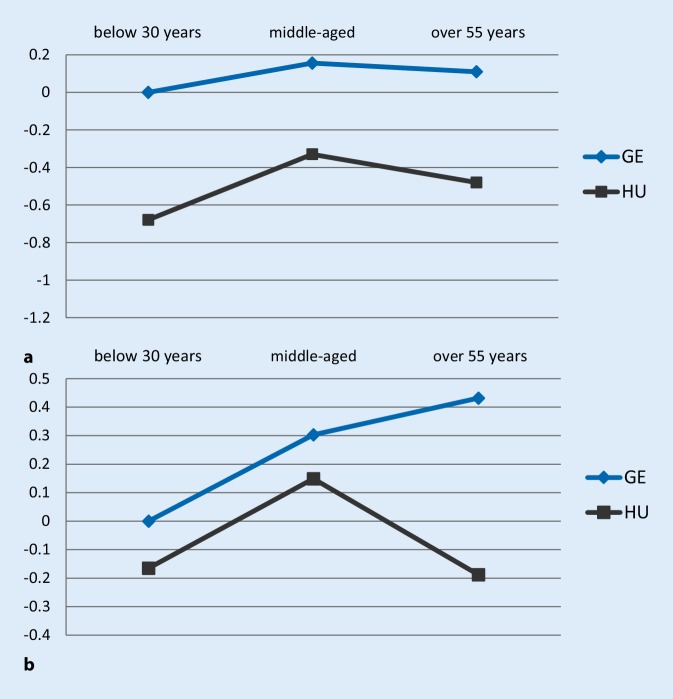
Job satisfaction-age profile estimates using the 2015 wave, subjective health included, *GE* Germany, *HU* Hungary; the Y-axis shows the estimated coefficient of employees’ job satisfaction relative to German young workers. **a** Dependent variable: Are you satisfied with your main job? **b** Dependent variable: Does your job give you the feeling of work well-done?

The final set of estimates explored the impact of health on the job satisfaction. Therefore, the final specifications were estimated using the 2015 wave. The results are summarized by Fig. 5a, b ﻿and they are comparable to those presented by Figs. 3 and 4:

Comparing the results with and without individual subjective health, the most striking difference between the two specifications was the sensitivity of the overall job satisfaction measure. The decreasing job satisfaction-age profiles turned into upward sloping relationship after controlling for health. This was true for the Hungarian and German case; however, in Hungary only in comparison with the youngest age group. These results suggest that health status is an important determinant of overall job satisfaction. Turning to the results with the work well-done measure, there were no marked differences between the specifications with and without the health control variable. This is in line with the expectations describing the “work well-done” measure as being more closely related to the job and working conditions itself, and is less affected by external conditions compared to overall job satisfaction.

In sum, the regression analysis suggests that those employees who more or less voluntarily stay in the labor market were more satisfied with their job. Thus, they might be a potentially more productive part of the workforce. Not considering demographic changes, the results might also call for the revival of early retirement schemes; however, as outlined in the Introduction, this is unrealistic given the financial and labor market tensions of aging. Instead, this study highlights the importance of allowing and encouraging longer working lives. Promoting longer working by providing appropriate working conditions should receive particular attention. As Fig. 5a, b illustrate, health and job satisfaction are related. Therefore, health prevention measures might be an important tool to improve the willingness to work among older employees. Besides, the regression analysis indicates that financial satisfaction, good relationship to the management, career opportunities, a harmony between work and family life and having a creative job where own skills can be used have a strong positive relation to job satisfaction, while being exposed to physical inconveniencies, having a monotone job, being under time pressure have negative impacts.^3^

## Policy implications and conclusions

This paper analyzes the job satisfaction-age profile of German and Hungarian employees with a focus on older vs. prime-aged workers using the EWCS. According to the results, less stringent pension regulations offering accessible pathways to early retirement and lower statutory retirement age provide the opportunity for older workers to self-select themselves into employment. Therefore, older employees in such regimes are more likely to voluntarily stay on the labor market and show a higher level of job satisfaction compared to workers living in countries with more stringent pension rules. This may have important consequences on firm-level productivity, employee-level workability, well-being, and even national pension and health care cost burdens. The restriction of early retirement pathways and the gradual introduction of higher statutory retirement ages constitute a necessary and key element of the pension policies in aging societies. Therefore, it would be of vital importance to improve the job satisfaction of older employees alongside to improving their employment participation rate.

This analysis has the following important policy messages. First, it would be crucial to give the opportunity for older employees to work if they voluntarily opt for working longer. They seem to be an especially motivated pool of employees and could productively contribute to decreasing the financial burdens caused by the demographic change. Pension regulations should include financial incentives to work longer. Indeed, initiatives both at the national and company level could be implemented to encourage workers to stay voluntarily longer on the labor market. However, it is important to note that we do not argue for universally higher retirement ages, as it would possibly exacerbate other social and economic problems. The most important message is to maintain appropriate incentive and motivation for those older employees who are willing to work. An important step towards this aim could be to improve the willingness to work among older employees by providing attractive working conditions and focusing on the improvement of their health.

## Caption Electronic Supplementary Material

Regression results including a description of the model specification

## Footnotes

1 The gap between the preferred and expected retirement age in Germany has been analyzed by Hess [14].

2 Using 55 years as the cut-off point is partly explained by technical reasons. The sample sizes by country did not allow a defininition of older workers using later age as a cut-off. The cut-off point of 55 years was often used in previous papers [13]. Additionally, the earliest age when people tended to retire before 2011 was 55 years in Hungary, at least for women.

3 As the rest of the coefficient estimates are restricted to be the same across countries and across age groups, this paper does not provide a detailed analysis about the importance of specific working conditions in determining job satisfaction; however, estimated coefficients on these control variables can be interpreted as average effects, and may serve as a coarse indicator on the relevance of working conditions in determining employees’ job satisfaction.
