# Supplementary material for: Job satisfaction at older ages: A comparative analysis of Hungarian and German data
Source: Z Gerontol Geriatr. 2019 Apr 26;53(1):44–50. doi: 10.1007/s00391-019-01547-x (PMC6985060; doi:10.1007/s00391-019-01547-x)
Supplement: Supplementary file 1 — Regression results including a description of the model specification [file 391_2019_1547_MOESM1_ESM.docx]

**Appendix 1: Model specification**

Ordered logistic regression models are estimated using the following specification:

(*i*) ${satisfaction}_{it}= \propto+ \beta_{1}{age30}_{it}H_{it}+\beta_{2}{age30\_55}_{it}+ \beta_{3}{age30\_55}_{it}H_{it}+\beta_{4}{age55}_{it}+ \beta_{5}{age55}_{it}H_{it}+ \nabla X_{it}+ \varepsilon_{it}$

In the equation above the age group dummies are denoted by *age30* (aged below 30), *age30_55* (aged between 30 and 55) and *age55* (aged over 55), *H* is the Hungarian country dummy, *i* refers to the person, *t* refers to time, and the matrix *X* includes the rest of the explanatory variables. The youngest age group without interaction (German workers aged below 30) serves as the reference category, therefore, it is not included into the regression. The interpretation of the age group coefficients is as follows: β_1_, β_3_ and β_5_ show the between country difference in the job satisfaction of each age group. For example, a negative and significant coefficient estimate on any of the interaction terms would imply that Hungarian employees in that age group are less satisfied than similarly aged German employees. However, the aim is not to examine the between country differences in job satisfaction, but to compare the relative job satisfaction of old vs. prime-aged employees. To test whether Hungarian workers over 55 are more satisfied with their jobs than Hungarian prime aged, then β_5_+ β_4_ and β_3_+ β_2_ need to be compared. The similar test in the case of German workers compares the coefficients β_4_ and β_2_. The direct implication of the hypothesis would be that (β_5_+ β_4_) – (β_3_+ β_2_) differs from β_4_ - β_2_, i.e. the relative job satisfaction of older workers is different in the two countries.

The final model specifications include several control variables, which are summarized below.

| **List of control variables** | **Dependent variables: How satisfied are you overall with your job? /**  **Do you have the feeling of work well-done?** |
| --- | --- |
| **(1)** | year |
| **(2)** | gender, education, working hours, commuting time, a dummy for self-employment, occupation, industry |
| **(4)** | financial satisfaction |
| **(5)** | personnel atmosphere (relationship with management, assistance from colleagues, good friends at the organization) |
| **(6)** | job security |
| **(7)** | career prospects |
| **(8)** | harmony with health (if the employee’s health or safety is in danger during work, if the employee is exposed to psychical inconveniences, such as noise or vibration, if the employee has to carry out physically demanding heavy work) |
| **(9)** | harmony with private life (if the job fits in with family and social commitments) |
| **(10)** | stress factors in the job (monotone work, presence of tight deadlines, work at a high speed work under time pressure) |
| **(11)** | opportunities for skill use (use own skills, meeting precise quality standards) |
| **(12)** | some other firm-level variables (firm size, training possibilities, discrimination, abuse) |
| **(13)** | subjective health status |

**Appendix 2: Computed coefficient estimates of the relative job satisfaction of employees**

|  |  | **2005-2010** | | | |
| --- | --- | --- | --- | --- | --- |
|  |  | ***Specification (1)*** | | ***Specification (2)*** | |
|  |  | **Basic: only year effects** | | **+ detailed demographic, firm and occupational variables** | |
|  |  | **overall job satisfaction** | **feeling of work well-done** | **overall job satisfaction** | **feeling of work well-done** |
| Job satisfaction relative to German young workers | below30 [GE] | reference | | reference | |
|  | middle-aged [GE] | -0.00709 | 0.303 | -0.137 | 0.216 |
|  | *p-value* | *0.000* | *0.000* | *0.000* | *0.000* |
|  | over55 [GE] | -0.0109 | 0.289 | -0.233 | 0.161 |
|  | *p-value* | *0.009* | *0.000* | *0.000* | *0.000* |
|  | below30 [HU] | -1.064 | -0.146 | -1.059 | -0.291 |
|  | *p-value* | *0.000* | *0.000* | *0.000* | *0.019* |
|  | middle-aged [HU] | -1.02409 | 0.192 | -1.052 | 0.042 |
|  | *p-value* | *0.000* | *0.000* | *0.000* | *0.400* |
|  | over55 [HU] | -0.8579 | 0.637 | -1.072 | 0.382 |
|  | *p-value* | *0.000* | *0.000* | *0.000* | *0.000* |
| Relative job satisfaction of older vs. prime aged | over 55 [HU] / middle-aged [HU] | 0.166 | 0.445 | -0.020 | 0.340 |
|  | *p-value* | *0.000* | *0.000* | *0.778* | *0.000* |
|  | over 55 [GE] / middle-aged [GE] | -0.004 | -0.014 | -0.096 | -0.055 |
|  | *p-value* | *0.320* | *0.085* | *0.000* | *0.042* |
| Test (Differences in relative job satisfaction) | | 0.000 | 0.000 | 0.097 | 0.001 |
| Observations | | 5,108 | 5,100 | 4,058 | 4,056 |

|  |  | **2015** | | | |
| --- | --- | --- | --- | --- | --- |
|  |  | ***Specification (2)*** | | ***Specification (3)*** | |
|  |  | **+ detailed demographic, firm and occupational variables** | | **Specification (2) +health status** | |
|  |  | **overall job satisfaction** | **feeling of work well-done** | **overall job satisfaction** | **feeling of work well-done** |
| Job satisfaction relative to German young workers | below30 [GE] | reference | | reference | |
|  | middle-aged [GE] | -0.164 | 0.191 | 0.156 | 0.303 |
|  | *p-value* | *0.049* | *0.000* | *0.130* | *0.000* |
|  | over55 [GE] | -0.406 | 0.255 | 0.11 | 0.432 |
|  | *p-value* | *0.000* | *0.000* | *0.205* | *0.000* |
|  | below30 [HU] | -0.665 | -0.171 | -0.679 | -0.165 |
|  | *p-value* | *0.000* | *0.000* | *0.000* | *0.000* |
|  | middle-aged [HU] | -0.579 | 0.059 | -0.33 | 0.149 |
|  | *p-value* | *0.000* | *0.000* | *0.065* | *0.000* |
|  | over55 [HU] | -0.944 | -0.341 | -0.480 | -0.188 |
|  | *p-value* | *0.000* | *0.000* | *0.003* | *0.190* |
| Relative job satisfaction of older vs. prime aged | over 55 [HU] / middle-aged [HU] | -0.365 | -0.400 | -0.150 | -0.337 |
|  | *p-value* | *0.000* | *0.000* | *0.000* | *0.003* |
|  | over 55 [GE] / middle-aged [GE] | -0.242 | 0.064 | -0.046 | 0.129 |
|  | *p-value* | *0.000* | *0.240* | *0.004* | *0.001* |
| Test (Differences in relative job satisfaction) | | 0.000 | 0.001 | 0.000 | 0.002 |
| Observations | | 1,930 | 1,927 | 1,930 | 1,927 |

*Notes: Appendix A2 includes the computed coefficient estimates of the relative job satisfaction of employees over 55. The estimates are computed from our ordered logistic regressions. Stars indicate significance levels: ***p<0.01, **p<0.05, *p<0.1. The row “Test (diff)” includes the p-value of the null hypothesis that “The difference between the job satisfaction of over 55 and prime-aged employees do not differ between the two countries”.*

**Appendix A3: Full set of estimated coefficients**

|  | **Dependent variable** | | | |
| --- | --- | --- | --- | --- |
|  | **2005-2010** | | **2005** | |
| **Controls** | **overall job satisfaction** | **feeling of work well-done** | **overall job satisfaction** | **feeling of work well-done** |
| aged below 30 * H | -1.059*** | -0.291** | -0.665*** | -0.171*** |
|  | *(0.0441)* | *(0.124)* | *(0.0799)* | *(0.00734)* |
| aged 30-55 | -0.137*** | 0.216*** | -0.164** | 0.191*** |
|  | *(0.0288)* | *(0.0258)* | *(0.0832)* | *(0.0475)* |
| aged 30-55 * H | -0.915*** | -0.174** | -0.415*** | -0.132*** |
|  | *(0.0323)* | *(0.0760)* | *(0.0595)* | *(0.0367)* |
| aged over 55 | -0.233*** | 0.161*** | -0.406*** | 0.255*** |
|  | *(0.0542)* | *(0.00144)* | *(0.0698)* | *(0.00726)* |
| aged over 55 * H | -0.839*** | 0.221*** | -0.538*** | -0.596*** |
|  | *(0.0781)* | *(0.0252)* | *(0.0711)* | *(0.104)* |
| female dummy | 0.146* | 0.136*** | -0.0508 | 0.0372 |
|  | *(0.0834)* | *(0.0116)* | *(0.179)* | *(0.0443)* |
| commuting time | -0.0023*** | 0.00098 | -0.0017*** | 0.00069 |
|  | *(0.000130)* | *(0.000617)* | *(0.000112)* | *(0.00251)* |
| weekly working hours | 0.0104*** | 0.00578*** | -0.00820 | 0.00489 |
|  | *(0.00191)* | *(0.000947)* | *(0.00583)* | *(0.00666)* |
| self employed | 0.739*** | 1.297*** | 1.091*** | 2.070*** |
|  | *(0.239)* | *(0.0508)* | *(0.268)* | *(0.228)* |
| highest educ level: secondary | -0.00518 | 0.404** | -0.350 | -0.696 |
|  | *(0.110)* | *(0.200)* | *(0.234)* | *(0.484)* |
| highest educ level: postsecondary | -0.447*** | 0.285 | -0.379*** | -1.264*** |
|  | *(0.0207)* | *(0.442)* | *(0.0588)* | *(0.285)* |
| highest educ level: tertiary | -0.126 | 0.331 | -0.691** | -1.118*** |
|  | *(0.0896)* | *(0.239)* | *(0.285)* | *(0.149)* |
| financial satisfaction | 1.149*** | 0.285*** | 1.252*** | 0.664*** |
|  | *(0.0821)* | *(0.0160)* | *(0.00997)* | *(0.0390)* |
| relationship with management | 0.410*** | 0.608*** | 1.304*** | 1.016*** |
|  | *(0.0943)* | *(0.0296)* | *(0.278)* | *(0.281)* |
| assistance from boss or colleagues | 0.636*** | 0.436*** | 0.406*** | 0.272 |
|  | *(0.0963)* | *(0.0354)* | *(0.157)* | *(0.262)* |
| career prospects | 0.663*** | 0.633*** | 0.827*** | 0.631*** |
|  | *(0.131)* | *(0.163)* | *(0.0849)* | *(0.0657)* |
| job security | 0.618*** | 0.454*** | -1.047*** | -0.518*** |
|  | *(0.0751)* | *(0.0403)* | *(0.0213)* | *(0.0308)* |
| health or safety is at risk | -0.706** | 0.0264 | -1.202*** | -0.0178 |
|  | *(0.306)* | *(0.0824)* | *(0.138)* | *(0.0172)* |
| exposed to physical inconveniences | -0.322*** | -0.0610 | -0.469*** | -0.190*** |
|  | *(0.0899)* | *(0.0790)* | *(0.0354)* | *(0.0422)* |
| physically demanding heavy work | -0.331*** | 0.0207 | 0.157 | -0.0477 |
|  | *(0.128)* | *(0.203)* | *(0.214)* | *(0.442)* |
| job fits in with family and social commitments | 0.636*** | 0.424*** | 0.675*** | 0.269 |
|  | *(0.0296)* | *(0.148)* | *(0.0183)* | *(0.219)* |
| monotonous job | -0.257*** | -0.457*** | -0.270*** | -0.439*** |
|  | *(0.0966)* | *(0.0123)* | *(0.0453)* | *(0.00612)* |
| tight deadlines or work at high speed | -0.404** | -0.357*** | -0.227*** | -0.498*** |
|  | *(0.160)* | *(0.0229)* | *(0.0119)* | *(0.0910)* |
| time pressure | -0.421*** | -0.571*** | -0.306*** | -0.535*** |
|  | *(0.0677)* | *(0.0191)* | *(0.0816)* | *(0.108)* |
| meeting precise quality standards | 0.0245*** | 0.381*** | 0.105 | 0.727*** |
|  | *(0.00739)* | *(0.0454)* | *(0.182)* | *(0.0379)* |
| using own skills | 0.353*** | 0.645*** | 0.379*** | 0.361* |
|  | *(0.124)* | *(0.131)* | *(0.00768)* | *(0.200)* |
| training | 0.0334 | -0.0156 | 0.144 | 0.0773*** |
|  | *(0.108)* | *(0.107)* | *(0.104)* | *(0.0253)* |
| firm size: medium | -0.251* | -0.157 | -0.204 | 0.00391 |
|  | *(0.141)* | *(0.152)* | *(0.125)* | *(0.149)* |
| firm size: large | -0.281** | -0.288*** | 0.0672 | -0.165 |
|  | *(0.117)* | *(0.0365)* | *(0.0458)* | *(0.106)* |
| exposed to discrimination | -0.593*** | -0.159*** | -0.435** | -0.687*** |
|  | *(0.175)* | *(0.0315)* | *(0.217)* | *(0.149)* |
| exposed to abuse | -0.825*** | -0.502*** | -0.725*** | -0.0241 |
|  | *(0.0977)* | *(0.0975)* | *(0.0126)* | *(0.0660)* |
| Observations | 4,058 | 4,056 | 1,930 | 1,927 |
| R-squared | 0.210 | 0.106 | 0.245 | 0.127 |

*Notes. Standard errors in parenthesis, stars indicate significance levels: *** p<0.01, ** p<0.05, * p<0.1. Standard errors clustered by country. Reference categories: aged below 30, highest education level: primary, firm size: small. The regression in specification (12) includes dummies for industry (6 categories), occupation (10 categories) and year. H is the Hungarian country dummy. Health status not included among the controls.*
